# Impact of *LINC00312* gene polymorphism coupled with habitual risks on buccal mucosa cancer

**DOI:** 10.7150/jca.94187

**Published:** 2024-03-04

**Authors:** Hui-Ting Hsu, Yen-Ting Lu, Yi-Tzu Chen, Ming-Tai Hsing, Chun-Wen Su, Shih-Chi Su, Chiao-Wen Lin, Shun-Fa Yang

**Affiliations:** 1 Institute of Medicine, Chung Shan Medical University, Taichung, Taiwan.; 2 School of Medicine, China Medical University, Taichung, Taiwan.; 3 Department of Pathology, China Medical University Hospital, Taichung, Taiwan.; 4 School of Medicine, Chung Shan Medical University, Taichung, Taiwan.; 5 Department of Otolaryngology, Chung Shan Medical University Hospital, Taichung, Taiwan.; 6 Department of Otolaryngology, St. Martin De Porres Hospital, Chiayi, Taiwan.; 7 Institute of Oral Sciences, Chung Shan Medical University, Taichung, Taiwan.; 8 Department of Dentistry, Chung Shan Medical University Hospital, Taichung, Taiwan.; 9 Department of Neurosurgery, Show Chwan Memorial Hospital, Changhua, Taiwan.; 10 Department of Medical Research, Chung Shan Medical University Hospital, Taichung, Taiwan.; 11 Whole-Genome Research Core Laboratory of Human Diseases, Chang Gung Memorial Hospital, Keelung, Taiwan.; 12 Department of Medical Biotechnology and Laboratory Science, College of Medicine, Chang Gung University, Taoyuan, Taiwan.

**Keywords:** *LINC00312*, genetic variants, OSCC, chewed betel quid

## Abstract

Oral squamous cell carcinoma (OSCC) is a prevalent and lethal malignancy with a diverse etiology. LINC00312 is a long intergenic non-coding RNA that functions as a signal hub to regulate the progression and treatment of head and neck cancer. The aim of this study was to evaluate the effect of *LINC00312* single nucleotide polymorphisms (SNPs) on the development of oral cancer. Two *LINC00312* SNPs, namely rs12497104 and rs164966, were investigated among 469 male patients with cancer of buccal mucosa and 1194 gender- and age-matched controls. No significant correlation was observed between these two SNPs and the occurrence of OSCC in the case and control groups. While assessing the clinicopathological features, carriers of at least one minor allele of rs164966 (GA and GG) were less prone to develop lymph node metastasis (adjusted odds ratio [AOR], 0.666; 95% confidence interval [CI], 0.447-0.991; *p*=0.045) in comparison with homozygous carriers of the major allele (AA). Subsequent stratifying surveys revealed that this genetic association with nodal spread was seen only in cases who habitually chewed betel quid (AOR, 0.616; 95% CI, 0.386-0.985; *p*=0.042) or smoked cigarettes (AOR, 0.612; 95% CI, 0.393-0.953; *p*=0.029), but undetected in cases free of these main behavioral risks. Our results indicate an interactivity of *LINC00312* rs164966 with lifestyle-related risks on modulating OSCC progression.

## Introduction

Oral oncogenesis, the transformation by which oral cancer develops, is a complex interplay of inherited genetic factors and environmental exposures 1. While a number of genetic factors contribute to genome stability, cell proliferation and programmed cell death 2, various environmental factors, such as viral infections 3 and chronic use of cigarette, alcohol, and betel quid (one areca nut-related product) 4, have been identified as significant risk factors for oral cancer. Besides, additional potential contributors to oral cancer include periodontal diseases 5, and infectious and inflammatory conditions 6. These etiologies highlight the multifaceted character of oral cancer. Oral squamous cell carcinoma (OSCC) remains the most prevalent form of oral cancer and is linked with substantial death rates 7 regardless of the presence of contemporary therapeutic approaches. Given the high heterogeneity of OSCC pathogenesis, all of the aforementioned risk factors appear to be interrelated and play a role in determining OSCC incidence and prognosis. In addition, another imperative feature of OSCC that may account for its heterogenous nature is the presence of different anatomical sites, each represented by a unique set of tissue organization and viewed as a distinct biological entity 8. Notably, oral cancer in Taiwan occurs primarily in the buccal mucosa 9, as OSCC most commonly presents as tongue cancer in the western countries 10.

The latest breakthroughs in the understanding of long non-coding RNA (lncRNA) functions have revolutionized the scope of molecular genetics 11. Until now, a considerable number of lncRNAs are found to be linked to a myriad of disorders 12, 13, including oral cancer 13-16. Among these cancer-associated lncRNAs, LINC00312, long intergenic non-coding RNA 312, was reported to be significantly downregulated in nasopharyngeal carcinoma (NPC), and its levels were negatively correlated with tumor size but positively correlated with lymph node metastasis in NPC 17. Functional assessment revealed an inhibitory effect of LINC00312 on cancer cell proliferation via induction of cell cycle arrest in NPC 18. On the contrary, through upregulation of matrix metalloprotease-1 (MMP-1), a promotive role of LINC00312 in NPC invasion was documented 19. In addition to the development and progression of NPC, a functional involvement of LINC00312 in NPC treatment was also noted, as this tumor suppressor was shown to improve the efficacy of radiotherapy in a xenograft mouse model of NPC by suppressing the phosphorylation of DNA-PKcs, a DNA-dependent protein kinase that is central to DNA double strand break repair 20. These findings suggest that LINC00312, acting as a signal hub, is capable of orchestrating NPC progression and treatment.

Lately, research employing targeted gene methods has demonstrated a genetic link between single-nucleotide polymorphisms (SNPs) of *LINC00312* and various aspects of NPC prognosis. Specifically, *LINC00312* variants, rs15734 and rs164966, were associated with a decrease in the risks of developing NPC, as patients with rs12497104 polymorphisms showed a poorer overall survival 21, 22. In addition, association of rs12497104 and rs15734 with chemoradiotherapy-induced hematotoxicity in NPC was recently reported 23. As *LINC00312* was found to be implicated in the development of oral submucous fibrosis 24 and head and neck cancer 25, the impact of *LINC00312* SNPs together with lifestyle-related risks on OSCC is in need of clarification. In this study, we performed a case-control survey to determine the extent to which genetic variants of *LINC00312* are associated with the risk of oral cancer.

## Materials and Methods

### Subjects

This study was approved by the institutional review board of Chung Shan Medical University Hospital, Taichung, Taiwan (CS1-21151). This study enrolled 469 male patients diagnosed with cancer of buccal mucosa and 1194 cancer-free male controls, who did not report a history of cancer or any oral precancerous conditions, to investigate the influence of *LINC00312* variants on the development of OSCC. Informed written consent was collected from all participants, recruited between 2012 and 2022, during enrollment. Grading and staging of cancer were determined by using the American Joint Committee on Cancer (AJCC) TNM staging system 26. The control group included males who did not report a history of cancer or any oral precancerous conditions like oral submucous fibrosis, leukoplakia, erythroplakia, verrucous hyperplasia, among others. Age and environmental risk information (including the use of areca nuts, tobacco, and alcohol) was gathered from all subjects.

### Genotyping

Two loci located within the *LINC00312* gene (rs12497104 and rs164966) were chosen for analysis in this study on the basis of their putative association with nasopharyngeal carcinoma susceptibility 21, 23. The QIAamp DNA Blood Mini Kit (Qiagen) was used to extract genomic DNA from whole blood specimens. The allelic discrimination of these two *LINC00312* SNPs including rs12497104 (assay ID: C_11757485_10), and rs164966 (assay ID: C_8753508_10) were assessed using the TaqMan assay performed with an ABI StepOne™ Real-Time PCR System (Applied Biosystems). The resulting data were processed by SDS version 3.0 software from Applied Biosystems.

### Statistical analysis

Demography and environmental exposures were compared between patients and controls using Mann-Whitney U-test. Multiple logistic regression models followed by adjustment for possible confounders were used to examine the association of genotypic ratios with OSCC risk or clinical status. Calculations were performed with SAS software (v9.4, 2013; SAS Institute Inc.) The threshold for a difference or association was set at a *p* value of <0.05.

## Results

### Demographic characteristics of recruited buccal mucosa cancer patients

This study enrolled 469 male patients with squamous cell carcinoma of buccal mucosa and 1194 cancer-free males to examine the relationship between *LINC00312* polymorphisms and the development of oral tumorigenesis. Demographic and clinical characteristics of both cohorts were assessed (**Table 1**). To exclude potential confounding factors, cancer-free controls with matched age and gender were recruited. Significant discrepancies in the proportions of cigarette smoking, alcohol consumption, and betel quid chewing were found between the case and control groups, in accordance with findings from other reports performed in Central and Southeast Asia 7, 27. In the case group, nodal and distal metastatic disease occurred in 30.5% and 0.6% of patients, respectively. Overall, 81.9% of the tumors in our cohort were moderately or poorly differentiated.

### Impacts of *LINC00312* genetic polymorphisms on the buccal mucosa cancer incidence

To explore the potential correlation of *LINC00312* variants with the development of oral cancer, we genotyped two SNPs of *LINC00312* gene, rs12497104 and rs164966, in our study groups, and no deviation from Hardy-Weinberg equilibrium was found (p>0.05) in both cohorts. Distributions of both genotype and allele frequency for individual SNP were determined in cases and controls. For these two SNPs, no significant correlations of their polymorphisms with the risk of oral cancer were observed between cases and controls (**Table 2**).

### Impacts of *LINC00312* genetic polymorphisms on clinicopathologic features of the buccal mucosa cancer

In addition, we tested the relationship between genotypic ratios of *LINC00312* SNPs and clinical variables of the disease cohort. We demonstrated that OSCC cases possessing at least one minor allele (G) of rs164966 (GA and GG) were less prone to develop lymph node metastasis (AOR, 0.666; 95% CI, 0.447-0.991; *p*=0.045) in comparison with homozygotes for the major allele (AA) (**Table 3**). These data indicate a putative association of LINC00312 gene variations with the metastatic potential of buccal mucosa cancer. Since a genetic correlation of *LINC00312* rs164966 with nodal spread of OSCC was detected, we continued to test for a joint effect of rs164966 and three main lifestyle-related risks (cigarette smoking, betel quid chewing, and drinking alcohol) on clinical variables of buccal mucosa cancer. Among patients who habitually used areca nut-related products (betel quid chewer, n=337) or cigarettes (smoker, n=387), a significant association of *LINC00312* rs164966 genotypes (GA+GG) with a decreased tendency to develop lymph node metastasis was noted, as compared to those homozygous for the major allele (AA) (AOR, 0.616; 95% CI, 0.386-0.985; *p*=0.042, for betel quid users; **Table 4**) (AOR, 0.612; 95% CI, 0.393-0.953; *p*=0.029, for cigarette consumers; **Table 5**). Nevertheless, such combined effect was not observed in patients who were not habitually exposed to betel quid chewing or cigarette smoking (**Table 4-5**). These results suggest that repeated exposure to carcinogens derived from lifestyle-related risks, combined with *LINC00312* polymorphisms, may affect OSCC progression.

## Discussion

It is widely acknowledged that the processes of oral oncogenesis are influenced by a mixture of predisposing factors, both environmental and hereditary. In this study, a link between *LINC00312* rs164966 genotypes and a reduced tendency to develop lymph node metastasis was established, while no correlation was found between the genotypic variants and disease occurrence. Subsequent stratification demonstrated that the association between genotypic variants of rs164966 and decreased nodal spread was only observed in frequent consumers of betel quid or cigarettes but not in cases without these main etiological parameters. Our findings indicate that *LINC00312* rs164966, coupled with lifestyle-related risks, interactively influence the progression of oral cancer.

Dysregulation of LINC00312 has been observed in various types of malignancies. In nasopharyngeal 17, blabber 28, and hepatic tumor 29, a decrease in LINC00312 expression was previously documented. However, rather than downregulation, upregulation of LINC00312 in oral submucous fibrosis, a precancerous disorder in the oral cavity, was reported 24. Such fluctuation of LINC00312 expression levels seems to be, in part, regulated by major habitual risks of OSCC. One piece of evidence has shown a significant induction of LINC00312 in oral cancer from patients with the history of tobacco chewing 30. Another study demonstrated a reduction of LINC00312 expression levels in OSCC cell lines treated with arecoline, a key component of areca nut extract 25. Chewing of areca nut-related products is a common risk factor for oral cancer among men in Taiwan (80%) 31, along with tobacco and alcohol use, but is rare in western countries 32. As cigarette smoking is known as a major driver of carcinogenesis in head and neck cancers 33, 34, chewing of areca nut-related products has been strongly implicated in the development of OSCC through prolonged exposure of various areca nut-derived carcinogens 35. It is reported that nitrosation of arecoline, the most abundant alkaloid in areca nuts, could generate a myriad of nitrosamines, which have high affinity with DNA, proteins or other molecules to trigger carcinogenic responses 36. Furthermore, reactive oxygen species (ROS) are produced in great amounts in the oral cavity during areca nut chewing to mediate oxidative damages in the DNA of buccal mucosa tissues 37. These metabolically activated reactive species of BQ-derived carcinogens collectively can induce genotoxic events in humans, such as production of Safrole-like DNA adducts 38 or chromosome breaks 39, ultimately causing oral tumorigenesis. In the present study, the genetic association of *LINC00312* with OSCC progression was found only in frequent users of betel quid or cigarettes but not in those who had no access to these key etiological parameters. Our results, together with findings from other reports, suggest that chronic exposure of tobacco- and areca nut-related products amplifies the effect of *LINC00312* gene polymorphisms on OSCC metastasis likely through manipulation of LINC00312 expression levels.

In addition to altered expression, functionality of LINC00312 may be also switched due to polymorphic alleles. It has been reported that polymorphic alleles of *LINC00312* rs164966 were predicted to cause corresponding changes in the secondary structure of *LINC00312* RNA transcripts, subsequently creating or abolishing the binding sites for miR-136-5p and miR-6729-3p, respectively 21. Accumulative evidence has pointed out a key role of miR-136-5p in cancer prognosis and treatment 40. As proposed as a tumor suppressor, miR-136-5p functionally interacts with a variety of target genes, such as ROCK1 41, BCL2 42, or SMAD3 43, to interfere with cancer cell invasion and migration. These findings indicate that altered sponging activities of LINC00312 attributed to its polymorphic alleles may be involved in regulating the invasive potential of OSCC.

In Taiwan, there has been a five-fold increase in the incidence of OSCC in men 44. The age-standardized incidence rate for men and the ratio of men to women are among the highest in Asia 1, 45. To eliminate one of potential confounders, the gender, only male subjects were included in this study. Another imperative feature of tumors in the oral cavity is the presence of different anatomical sites, which are characterized by unique sets of tissue organization and considered as different biological entities 8. The presence of high site-specific heterogeneity in molecular expression 46-48 could very likely impact the cancer development and precision medicine. It is noteworthy that oral cancer in Taiwan most commonly occurs in the buccal mucosa 9, accounting for approximately 40% of cases. However, in western countries, carcinoma of buccal mucosa is significantly less common, as OSCC primarily presents as tongue cancer in the West 10. These regional differences and lncRNA landscape disparities highlight the long-standing notion that Asian OSCC, particularly those caused by betel quid chewing, is a distinct disease from those in the West.

The present investigation revealed a synergistic effect of *LINC00312* gene polymorphisms and behavioral risk parameters on lymph node metastasis of OSCC. However, further explorations are necessary to address certain limitations of this work. One such limitation is the absence of quantitative definitions for key behavioral risks like the use of betel nuts, alcohol, and cigarettes. This could lead to an underestimation of these etiologic factors on affecting OSCC progression. Another concern is that the mechanistic roles of *LINC00312* rs164966 in orchestrating nodal spread remain unclear, as the expression levels of this gene have been observed to be positively correlated with lymph node metastasis in NPC 17. Additional investigations are needed to determine whether *LINC00312* rs164966 serves as a quantitative trait locus to modulate its own expression or alters the hub activities to perturb its affinity to specific microRNAs. Furthermore, our results might only apply to certain ethnic groups and require replication in additional study cohorts.

In conclusion, our data demonstrate a correlation between *LINC00312* rs164966 and the frequency of developing nodal spread in cancer of buccal mucosa. This genetic association was only found in cases who habitually consumed betel quid or cigarettes, and undetected in those who were not exposed to these significant environmental risks. These results indicate that repeated access to carcinogens derived from lifestyle-related risks, interacted with *LINC00312* polymorphisms, may affect the metastatic potential of oral cancer.

## Figures and Tables

**Table 1:** Comparisons of clinical and demographic characteristics in male patients with cancers of the buccal mucosa (n=469) and cancer-free controls (n=1194).

Variable	Controls (N=1194)	Patients (N=469)	*p* value
Age (yrs)			
< 55	564 (47.2%)	214 (45.6%)	*p* = 0.554
≥ 55	630 (52.8%)	255 (54.4%)	
Betel quid chewing			
No	996 (83.4%)	132 (28.1%)	
Yes	198 (16.6%)	337 (71.9%)	*p* < 0.001*
Cigarette smoking			
No	559 (46.8%)	82 (17.5%)	
Yes	635 (53.2%)	387 (82.5%)	*p* < 0.001*
Alcohol drinking			
No	957 (80.2%)	270 (57.6%)	
Yes	237 (19.8%)	199 (42.4%)	*p* < 0.001*
Stage			
I+II		224 (47.8%)	
III+IV		245 (52.2%)	
Tumor T status			
T1+T2		236 (50.3%)	
T3+T4		233 (49.7%)	
Lymph node status			
N0		326 (69.5%)	
N1+N2+N3		143 (30.5%)	
Metastasis			
M0		466 (99.4%)	
M1		3 (0.6%)	
Cell differentiation			
Well differentiated		85 (18.1%)	
Moderately or poorly differentiated		384 (81.9%)	

Mann-Whitney U test was used between controls and male patients with cancers of the buccal mucosa. * *p* value < 0.05 as statistically significant.

**Table 2:** Association of *LINC00312* genotypes/alleles with the risk of male patients with cancers of the buccal mucosa.

Variable	Controls (N=1194) (%)	Patients (N=469) (%)	AOR (95% CI)^a^
rs12497104			
GG	477 (39.9%)	180 (38.4%)	1.000 (reference)
GA	554 (46.4%)	229 (48.8%)	1.084 (0.826-1.424)
AA	163 (13.7%)	60 (12.8%)	0.932 (0.622-1.395)
GA+AA	717 (60.1%)	289 (61.6%)	1.049 (0.810-1.359)
G allele	1508 (63.1%)	589 (62.8%)	1.000 (reference)
A allele	880 (36.9%)	349 (37.2%)	0.996 (0.828-1.199)
**rs164966**			
AA	662 (55.4%)	246 (52.5%)	1.000 (reference)
AG	456 (38.2%)	191 (40.7%)	1.068 (0.819-1.392)
GG	76 (6.4%)	32 (6.8%)	1.033 (0.615-1.736)
AG+GG	532 (44.6%)	223 (47.5%)	1.063 (0.824-1.370)
A allele	1780 (74.5%)	683 (72.8%)	1.000 (reference)
G allele	608 (25.5%)	255 (27.2%)	1.040 (0.850-1.274)

^a^ The adjusted odds ratio (AOR) with their 95% confidence intervals were estimated by multiple logistic regression models after controlling for betel quid chewing, cigarette smoking, and alcohol drinking.

**Table 3:** Clinical statuses and *LINC00312* rs12497104 and rs164966 genotype frequencies in male patients with cancers of the buccal mucosa.

	rs12497104 (N=469)				rs164966 (N=469)			
Variable	GG(N=180)	GA+AA(N=289)	AOR (95% CI)	*p* value	AA(N=246)	AG+GG(N=223)	AOR (95% CI)	*p* value
**Clinical Stage**								
Stage I+II	80 (44.4%)	144 (49.8%)	1.000 (reference)	0.256	114 (46.3%)	110 (49.3%)	1.000 (reference)	0.518
Stage III+IV	100 (55.6%)	145 (50.2%)	0.806 (0.554-1.170)		132 (53.7%)	113 (50.7%)	0.887 (0.617-1.275)	
**Tumor size**								
≦ T2	88 (48.9%)	148 (51.2%)	1.000 (reference)	0.625	121 (49.2%)	115 (51.6%)	1.000 (reference)	0.606
> T2	92 (51.1%)	141 (48.8%)	0.911 (0.628-1.322)		125 (50.8%)	108 (48.4%)	0.909 (0.633-1.306)	
**Lymph node metastasis**								
No	123 (68.3%)	203 (70.2%)	1.000 (reference)	0.662	161 (65.4%)	165 (74.0%)	1.000 (reference)	0.045*
Yes	57 (31.7%)	86 (29.8%)	0.914 (0.611-1.368)		85 (34.6%)	58 (26.0%)	0.666 (0.447-0.991)	
**Metastasis**								
M0	179 (99.4%)	287 (99.3%)	1.000 (reference)	0.857	244 (99.2%)	222 (99.6%)	1.000 (reference)	0.621
M1	1 (0.6%)	2 (0.7%)	1.247 (0.112-13.856)		2 (0.8%)	1 (0.4%)	0.550 (0.049-6.102)	
**Cell differentiation**								
Well	32 (17.8%)	53 (18.3%)	1.000 (reference)	0.878	44 (17.9%)	41 (18.4%)	1.000 (reference)	0.888
Moderate or poor	148 (82.2%)	236 (81.7%)	0.963 (0.593-1.563)		202 (82.1%)	182 (81.6%)	0.967 (0.604-1.547)	

Cell differentiation grade: grade I, well differentiated; grade II, moderately differentiated; grade III, poorly differentiated. The adjusted odds ratio (AOR) with their 95% confidence intervals were estimated by multiple logistic regression models after controlling for cigarette smoking, and alcohol drinking. * *p* value < 0.05 as statistically significant.

**Table 4:** Clinical statuses and genotypic frequencies of *LINC00312* rs164966 in buccal mucosa cancer who are betel quid chewers or not betel quid chewers.

	Betel Quid Chewers (N=337)				Non-Betel Quid Chewers (N=132)			
Variable	AA(N=173)	AG+GG(N=164)	AOR (95% CI)	*p* value	AA(N=73)	AG+GG(N=59)	AOR (95% CI)	*p* value
**Clinical Stage**								
Stage I+II	78 (45.1%)	81 (49.4%)	1.000 (reference)	0.429	36 (49.3%)	29 (49.2%)	1.000 (reference)	0.985
Stage III+IV	95 (54.9%)	83 (50.6%)	0.841 (0.548-1.291)		37 (50.7%)	30 (50.8%)	1.007 (0.507-1.999)	
**Tumor size**								
≦ T2	82 (47.4%)	81 (49.4%)	1.000 (reference)	0.715	39 (53.4%)	34 (57.6%)	1.000 (reference)	0.629
> T2	91 (52.6%)	83 (50.6%)	0.923 (0.602-1.416)		34 (46.6%)	25 (42.4%)	0.843 (0.422-1.684)	
**Lymph node metastasis**								
No	111 (64.2%)	122 (74.4%)	1.000 (reference)	0.042*	50 (68.5%)	43 (72.9%)	1.000 (reference)	0.583
Yes	62 (35.8%)	42 (25.6%)	**0.616 (0.386-0.985)**		23 (31.5%)	16 (27.1%)	0.809 (0.379-1.725)	
**Metastasis**								
M0	172 (99.4%)	163 (99.4%)	1.000 (reference)	0.970	72 (98.6%)	59 (100.0%)	1.000 (reference)	0.367
M1	1 (0.6%)	1 (0.6%)	1.055 (0.065-17.010)		1 (1.4%)	0 (0.0%)	---	
**Cell differentiation**								
Well	37 (21.4%)	34 (20.7%)	1.000 (reference)	0.883	7 (9.6%)	7 (11.9%)	1.000 (reference)	0.673
Moderate or poor	136 (78.6%)	130 (79.3%)	1.040 (0.616-1.757)		66 (90.4%)	52 (88.1%)	0.788 (0.260-2.388)	

Cell differentiation grade: grade I, well differentiated; grade II, moderately differentiated; grade III, poorly differentiated. The adjusted odds ratio (AOR) with their 95% confidence intervals were estimated by multiple logistic regression models after controlling for cigarette smoking, and alcohol drinking. * *p* value < 0.05 as statistically significant.

**Table 5:** Clinical statuses and genotypic frequencies of *LINC00312* rs164966 in buccal mucosa patients who are cigarette smokers or not cigarette smokers.

	Cigarette Smokers (N=387)				Non-Cigarette Smokers (N=82)			
Variable	AA(N=209)	AG+GG(N=178)	AOR (95% CI)	*p* value	AA(N=37)	AG+GG(N=45)	AOR (95% CI)	*p* value
**Clinical Stage**								
Stage I+II	95 (45.5%)	92 (51.7%)	1.000 (reference)	0.222	19 (51.4%)	18 (40.0%)	1.000 (reference)	0.304
Stage III+IV	114 (54.5%)	86 (48.3%)	0.779 (0.522-1.163)		18 (48.6%)	27 (60.0%)	1.583 (0.658-3.811)	
**Tumor size**								
≦ T2	103 (49.3%)	97 (54.5%)	1.000 (reference)	0.306	18 (48.6%)	18 (40.0%)	1.000 (reference)	0.432
> T2	106 (50.7%)	81 (45.5%)	0.811 (0.544-1.211)		19 (51.4%)	27 (60.0%)	1.421 (0.590-3.420)	
**Lymph node metastasis**								
No	136 (65.1%)	134 (75.3%)	1.000 (reference)	0.029*	25 (67.6%)	31 (68.9%)	1.000 (reference)	0.898
Yes	73 (34.9%)	44 (24.7%)	**0.612 (0.393-0.953)**		12 (32.4%)	14 (31.1%)	0.941 (0.370-2.394)	
**Metastasis**								
M0	207 (99.0%)	177 (99.4%)	1.000 (reference)	0.659	37 (100.0%)	45 (100.0%)	1.00 (reference)	----
M1	2 (1.0%)	1 (0.6%)	0.585 (0.053-6.503)		0 (0.0%)	0 (0.0%)	---	
**Cell differentiation**								
Well	38 (17.4%)	37 (20.8%)	1.000 (reference)	0.518	6 (16.2%)	4 (8.9%)	1.000 (reference)	0.313
Moderate or poor	171 (81.8%)	141 (79.2%)	0.847 (0.511-1.403)		31 (83.8%)	41 (91.1%)	1.984 (0.515-7.641)	

Cell differentiation grade: grade I, well differentiated; grade II, moderately differentiated; grade III, poorly differentiated. The adjusted odds ratio (AOR) with their 95% confidence intervals were estimated by multiple logistic regression models after controlling for betel quid chewing, and alcohol drinking. * *p* value < 0.05 as statistically significant.
